# Economic evaluation of atezolizumab in combination with bevacizumab and chemotherapy for metastatic, persistent, or recurrent cervical cancer in China: A cost-effectiveness analysis

**DOI:** 10.1371/journal.pone.0351029

**Published:** 2026-06-11

**Authors:** Shuo Yang, Yibing Hou, Xiaohui Wang, Shuo Kang, Zhenhua Pan

**Affiliations:** 1 School of Pharmacy, Hebei Medical University, Shijiazhuang, Hebei, People’s Republic of China; 2 Pharmacy, The Second Hospital of Hebei Medical University, Shijiazhuang, Hebei, People’s Republic of China; 3 Development Planning Division, Hebei Medical University, Shijiazhuang, Hebei, People’s Republic of China; 4 Development Planning Division, The Second Hospital of Hebei Medical University, Shijiazhuang, People’s Republic of China; University of Nigeria Teaching Hospital, NIGERIA

## Abstract

**Background:**

From China’s healthcare perspective, this analysis compared the cost-effectiveness of Atezolizumab in combination with Bevacizumab and chemotherapy for Metastatic, Persistent, or Recurrent Cervical Cancer.

**Methods:**

A Markov model was developed to track patients’ transitions over 3-week cycles and evaluate the health and economic outcomes over a 10-year horizon for the two competing treatments. The survival data were gathered from the BEATcc trial, and cost and utility values were obtained from the published studies. Total costs, life-years, quality-adjusted life-years (QALYs), and incremental cost-effectiveness ratio (ICER) were the model outcomes. Sensitivity analyzes were performed to examine the robustness of the model results.

**Results:**

In the base case, atezolizumab plus bevacizumab and chemotherapy yielded a marginal cost of $233,602.51 and an additional 0.54 QALYs, resulting in an ICER of $432,597.24 per additional QALY gained, which exceeded the willingness-to-pay (WTP) threshold of $36,859 in China. Sensitivity analyzes confirmed the robustness of the model outcomes.

**Conclusions:**

Atezolizumab plus bevacizumab and chemotherapy was not a cost-effective treatment for patients with metastatic, persistent, or recurrent cervical cancer compared with bevacizumab plus chemotherapy from the perspective of the Chinese health-care system.

## 1. Introduction

Cervical cancer is one of the most common gynecological malignancies and a leading cause of cancer-related mortality among women globally. Notably, China has the highest incidence of cervical cancer worldwide [1]. Most patients with early cervical cancer can be cured by surgical treatment, while patients with metastatic, persistent, or recurrent cervical cancer have limited treatment options [2]. Despite advancements in treatment, the prognosis of advanced cervical cancer remains dismal, with a 5-year survival rate of 17%, underscoring the unmet clinical need [3]. In addition to surgery, radiation therapy, chemotherapy and targeted therapy, immunotherapy has made great progress in patients with malignant tumors in recent years. Immune checkpoint inhibitors (ICIs) have brought more survival benefits to patients, which would enhance the anti-tumor immune function of T cells by inhibiting the immune checkpoint and ligand binding on the surface of patients’ immune cells, thus playing a role in killing tumor cells [4]. It is a novel treatment model for patients with metastatic, persistent, or recurrent cervical cancer.

Atezolizumab is a humanized IgG1 monoclonal antibody that targets PD-L1, which can relieve the inhibitory state of the tumor immune microenvironment and T cell exhaustion by blocking the binding between PD-L1 and its receptor PD-1, promote the activation and proliferation of tumor-specific T cells, and thus achieve the elimination of tumor cells [5]. Bevacizumab is a recombinant humanized IgG1 monoclonal antibody. By binding to vascular endothelial growth factor (VEGF), it inhibits the interaction between VEGF and its receptors, so as to achieve the effects of inhibiting tumor angiogenesis, growth and metastasis [6]. The efficacy and safety of atezolizumab plus bevacizumab and chemotherapy for the treatment of metastatic, persistent, or recurrent cervical cancer was demonstrated in BEATcc, a phase 3, global, multicenter RCT [7].

Jianying Lei et al. reported a cost-effectiveness analysis based on the BEATcc trial [8]. However, their study was directly compared from the United States healthcare payers’ perspective. Therefore, it is necessary to evaluate the cost-effectiveness of atezolizumab treatment from the Chinese perspective. The objective of our current study is to determine the cost-effectiveness of atezolizumab plus bevacizumab and chemotherapy compared with bevacizumab plus chemotherapy for patients with metastatic, persistent, or recurrent cervical cancer from the perspective of the Chinese health-care system.

## 2. Methods

### 2.1. Target population and interventions

The target population of this study was consistent with the patient inclusion criteria of the BEATcc clinical trial. Eligible patients were adults (>18 years of age) who met the following criteria: measurable metastatic (stage IVB), persistent, or recurrent cervical cancer according to the Response Evaluation Criteria in Solid Tumors version 1.1 (RECIST v1.1), not amenable to curative surgery or radiotherapy; histologically confirmed squamous cell carcinoma or adenocarcinoma; availability of archived or recently collected tumor tissue samples; and a Gynecologic Oncology Group (GOG) or Eastern Cooperative Oncology Group (ECOG) performance status of 0 or 1. Additional inclusion criteria included prior systemic therapy for metastatic, persistent, or recurrent disease; presence of active lesions involving the bladder or rectum; prior treatment with any anti‑vascular endothelial growth factor (VEGF) therapy or immune checkpoint inhibitors; and other factors associated with an increased risk of toxicity related to bevacizumab or atezolizumab [7].

In the BEATcc trial, enrolled patients were randomly assigned in a 1:1 ratio to receive either bevacizumab plus chemotherapy or atezolizumab plus bevacizumab plus chemotherapy. Patients in the atezolizumab group received intravenous atezolizumab 1200 mg every three weeks. Both treatment groups followed a three‑week cycle. Intravenous platinum (cisplatin 50 mg/m²), intravenous paclitaxel 175 mg/m², and intravenous bevacizumab 15 mg/kg were all administered on day 1 of each three‑week cycle. Treatment continued until disease progression, unacceptable toxicity, patient withdrawal, or death, whichever occurred first [7]. In accordance with the Clinical Diagnosis and Treatment Guidelines for Cervical Cancer (2024) and the BEATcc trial background, patients received a pembrolizumab‑based second‑line regimen following disease progression.

### 2.2. Analytical overview and model structure

**Fig 1:**
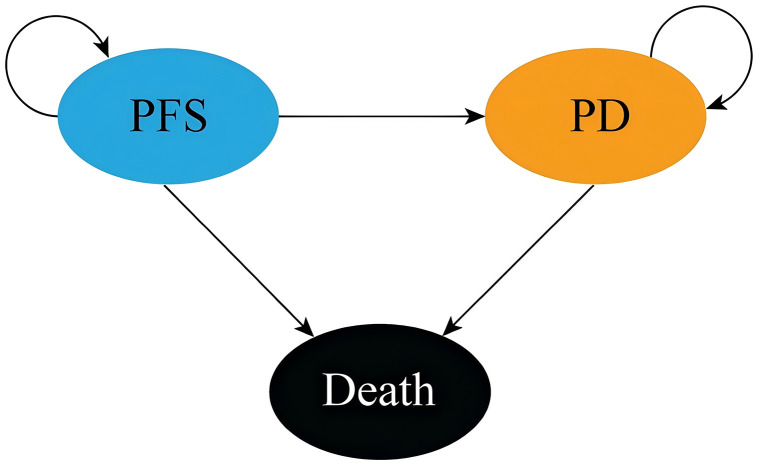
The structure of the Markov model. PFS, progression-free survival; PD, progressed disease.

A Markov model comprising three mutually exclusive health states—progression-free survival (PFS), progressive disease (PD), and death—was developed to estimate long-term costs and clinical outcomes (Fig 1). The model compared two first-line treatment strategies, both administered every three weeks: atezolizumab plus bevacizumab and chemotherapy versus bevacizumab plus chemotherapy. A 10-year time horizon was adopted. All patients entered the model in the PFD state. In each cycle, patients could remain progression-free, transition to PD, or die. The primary outcomes were total costs, quality-adjusted life-years (QALYs), and the incremental cost-effectiveness ratio (ICER). Costs and QALYs were discounted at an annual rate of 5% [9]. All costs are reported in 2024 U.S. dollars, converted at an exchange rate of US $1 = CNY 7.297. Consistent with World Health Organization recommendations, the willingness-to-pay threshold was set at three times China’s per capita gross domestic product in 2023, equivalent to $36,859 per QALY [10].

### 2.3. Clinical data input

Survival inputs for the model were derived from the BEATcc trial. Long-term survival estimates were generated by fitting parametric distributions to and extrapolating the trial’s progression-free survival (PFS) and overall survival (OS) Kaplan-Meier curves. The published PFS and OS curves were digitized using GetData Graph Digitizer software (version 2.26) to extract time-to-event data. Subsequently, parametric survival modeling was performed using R software (version 4.4.2). A range of standard distributions were evaluated, including exponential, gamma, Weibull, log-normal, log-logistic, Gompertz, and a Royston/Parmar spline model [11,12]. Model selection was guided by the Akaike information criterion (AIC), the Bayesian information criterion (BIC), and visual inspection of the fitted curves against the original Kaplan-Meier data [13], with lower AIC and BIC values indicating a superior fit [14]. The AIC values for all candidate models are presented in S1 Table, and the corresponding distribution parameters for each curve are summarized in Table 1.log-logistic survival function S(t) = 1/(1 + λt^γ^), Weibull and generalized gammasurvival functions were used to calculate the time-dependency transition probabilities.

**Table 1:** Survival model parameters fitting to the PFS and OS data from the BEATcc trial.

Treatment	PFS		OS	
	Model	parameters	Model	parameters
Bevacizumab plus chemotherapy	Log-logistic	Shape = 2.182 Scale = 11.329	Weibull	Shape = 1.553 Scale = 30.916
Atezolizumab plus bevacizumab and chemotherapy	Generalized gamma	Mu = 2.6088 Sigma = 1.0171	Log-logistic	Shape = 1.89 Scale = 30.55

PFS: progression-free survival, OS: overall survival.

The transition probability from the progression-free survival (PFS) state to death (pFTD) was assumed to be equivalent to the age-adjusted mortality rate of the general Chinese population. The probability of remaining in the PFS state in a subsequent cycle (pFTF) was calculated as pFTF = S(t)/ S(t-1), where S(t) denotes the overall survival probability at time t. The transition probability from PFS to progressive disease (PD), denoted as pFTP, was then derived as: pFTP = 1 − pFTD − pFTF. Similarly, the transition probability from overall survival (encompassing both PFS and PD states) to survival in the next cycle (pSTS) was estimated from the fitted parametric survival function. Subsequently, the probability of remaining in the PD state (pPTP) was calculated using the formula: pPTP = [(nPFS + nPD) × pSTS − (nPFS × pFTF) − (nPFS × pFTP)]/ nPD, where nPFS and nPD represent the number of patients in the PFS and PD states, respectively, at the end of the previous Markov cycle. Finally, the transition probability from PD to death (pPTD) was defined as: pPTD = 1 − pPTP.

### 2.4 Cost and utility values

This economic evaluation was conducted from the perspective of the Chinese healthcare system. Accordingly, only direct medical costs were considered, including expenses related to study drugs (atezolizumab and first-line chemotherapy agents), supportive care, routine follow-up, palliative treatment, and grade ≥3 adverse events for which the difference in incidence between the two groups was at least 5%. The incidence rates of grade ≥3 adverse events were obtained directly from the BEATcc trial protocol. For the calculation of drug dosages and associated costs, standard patient characteristics were applied: a body surface area of 1.72 m², a body weight of 65 kg [15]. Drug costs were based on provincial tender prices, while costs for other medical resources were derived from published literature. All costs were adjusted to 2023 values using China’s Consumer Price Index (CPI). Health-state utilities, measured on a scale from 0 (death) to 1 (perfect health), were applied to calculate quality-adjusted life-years (QALYs). The utility values assigned to the progression-free survival (PFS), progressive disease (PD), and death health states were 0.817, 0.779, and 0, respectively, based on published sources [8]. All relevant parameters and their assigned probability distributions are detailed in Table 2.

**Table 2:** Model inputs: baseline values and ranges for sensitivity analyzes.

Parameter	Baseline value	Range		Reference
		Upper bound	Lower bound	
Cost values (US $)				
Atezolizumab (1200 mg)	4507.8352	5409.40224	3606.26816	Local charge
Bevacizumab (100 mg)	154.7507	185.70084	123.80056	Local charge
Cisplatin (10 mg)	5.4699	6.56388	4.37592	Local charge
Paclitaxel (30 mg)	7.3733	8.84796	5.89864	Local charge
Pembrolizumab (0.1 g)	2462.5424	2955.05088	1970.03392	Local charge
Anemia	531.7	638.04	425.36	[16]
Neutropenia	399.63	479.556	319.704	[17]
Supportive treatment	274.63	329.556	219.704	[17]
Routine follow-up	55.6	66.72	44.48	[16]
Palliative treatment	685.9	823.08	548.72	[17]
Utility values				
PFS	0.817	0.9804	0.6536	[8]
PD	0.779	0.9768	0.6232	[8]
Others				
Discount rate	5.0	6	4	[9]
Probability of anemia occurrence in bevacizumab plus chemotherapy group	7.0	8.4	5.6	[18]
Probability of neutropenia occurrence in bevacizumab plus chemotherapy group	25	30	20	[18]
Probability of anemia occurrence in atezolizumab plus bevacizumab and chemotherapy group	14	16.8	11.2	[18]
Probability of neutropenia occurrence in atezolizumab plus bevacizumab and chemotherapy group	18	21.6	14.4	[18]
Proportion of patients in bevacizumab plus chemotherapy group who receive second – line treatment	58	69.6	46.4	[18]
Proportion of patients in atezolizumab plus bevacizumab and chemotherapy group who receive second – line treatment	54	64.8	43.2	[18]

PFS: progression-free survival, PD: progressed disease.

### 2.5. Sensitivity analyzes

Model robustness was assessed through both one-way and probabilistic sensitivity analyzes. In the one-way sensitivity analysis, the impact of individual parameter uncertainty on the incremental cost-effectiveness ratio (ICER) was evaluated by varying each parameter across its plausible range. These ranges were defined by the 95% confidence intervals from published sources or, where unavailable, by applying a ± 25% variation to the base-case value. The results of this analysis are presented in a tornado diagram [19]. For the probabilistic sensitivity analysis (PSA), a Monte Carlo simulation with 1,000 iterations was performed. Key model parameters were simultaneously sampled from their prespecified statistical distributions: gamma distributions for costs, and beta distributions for utility values and event probabilities. The results of the PSA are illustrated using cost-effectiveness scatterplots and cost-effectiveness acceptability curves (CEACs)

## 3. Results

### 3.1. Base case analysis

Based on the base-case results presented in Table 3, from the perspective of the Chinese health-care system and over a 10-year time horizon, the addition of atezolizumab to bevacizumab and chemotherapy was associated with an incremental gain of 0.54 QALYs at an additional cost of $233,602.51 compared with bevacizumab plus chemotherapy in patients with metastatic, persistent, or recurrent cervical cancer. This yielded an ICER of $432,597.24 per QALY, which far exceeded the WTPthreshold of $36,859/QALY in China. Accordingly, the atezolizumab regimen is unlikely to be considered cost‑effective in the Chinese setting.

**Table 3:** The results of base-case analyzes.

Treatment	Cost/$	QALYs	ICER($/QALY)
Atezolizumab plus bevacizumab and chemotherapy	329542.25	3.19	–
Bevacizumab plus chemotherapy	95939.74	2.65	–
Incremental	233602.51	0.54	432597.24

QALYs: quality-adjusted life-years, ICER: incremental cost-effectiveness ratio.

### 3.2. Sensitivity analyzes

**Fig 2:**
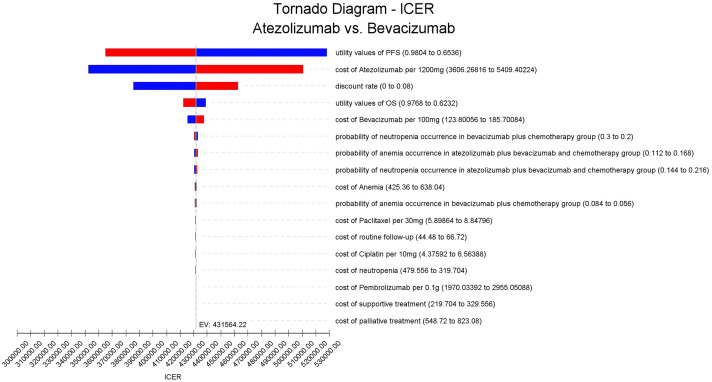
Tornado diagram of one-way sensitivity analyzes for patients. PFS, progression-free survival; PD, progressed disease.

**Fig 3:**
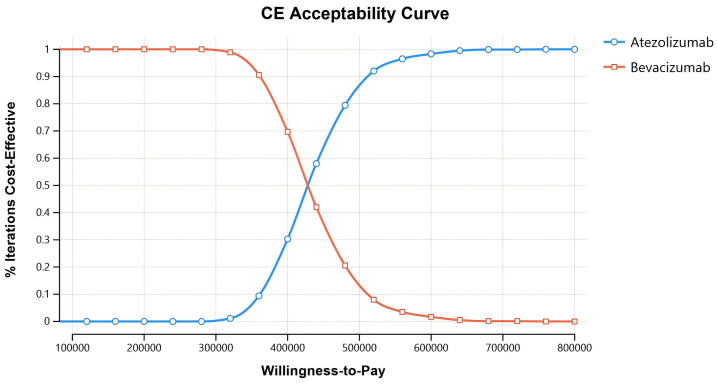
Cost-effectiveness acceptability curves for patients.

The tornado diagram derived from one-way sensitivity analyses identified the utility values for PFS and the cost of atezolizumab as the primary drivers of the ICER. Variation of the model parameters indicated that the results remained robust. PSA showed that, at China’s WTP threshold of $36,859 per QALY, the probability of the atezolizumab-containing regimen being cost-effective was 0%, further confirming the robustness of the model findings (Figs 2 and 3).

## 4. Discussion

In the BEATcc trial, atezolizumab plus bevacizumab and chemotherapy statistically extended PFS and OS in patients with metastatic, persistent, or recurrent cervical cancer compared with bevacizumab plus chemotherapy. However, the high cost of atezolizumab might bring a financial burden for the majority of middle- and low-income patients, it was uncertain about which treatment is more beneficial. Therefore, it is an urgent agenda to conduct a cost-effectiveness analysis to evaluate the efficacy and cost of atezolizumab combined therapy in the Chinese context. As far as we know, this is the first economic study on atezolizumab combined therapy for metastatic, persistent, or recurrent cervical cancer in China. Base-case analysis demonstrated that atezolizumab plus bevacizumab and chemotherapy yielded 0.54 additional QALYs at a marginal cost of $233,602.51, generating an ICER of $432,597.24/QALY—exceeding China’s willingness-to-pay (WTP) threshold. One-way sensitivity analysis identified progression-free survival utility and atezolizumab cost as primary ICER drivers. Probabilistic sensitivity analysis confirmed universal non-cost-effectiveness (100% probability) at China’s $36,859/QALY benchmark.

Previously, the phase 3 Gynaecologic Oncology Group 240 trial established chemotherapy in combination with bevacizumab as standard first-line treatment for incurable cervical cancer, with a median overall survival of approximately 17 months [20]. The Keynote-826 trial demonstrated that adding pembrolizumab to first-line chemotherapy could bring significant clinical benefits [21]. Up to now, immune checkpoint inhibitors including atezolizumab and pembrolizumab are available for the treatment of patients with metastatic, persistent, or recurrent cervical cancer, and there were studies evaluating their cost-effectiveness. For research about pembrolizumab, five studies showed that the addition of pembrolizumab in various combinations in patients with metastatic, persistent, or recurrent cervical cancer was unlikely to be cost-effective in both China and the US due to the high price of pembrolizumab [16,17,22–24]. Three studies demonstrated that adding atezolizumab to bevacizumab combined with chemotherapy was not a cost-effective option compared with bevacizumab plus chemotherapy in the United States [8,25,26]. Evidence suggests pembrolizumab and atezolizumab combination therapy is unlikely to be cost-effective for metastatic/recurrent cervical cancer in either high-income economies like the United States or resource-constrained settings like China. Although they could prolong the survival period of patients and bring more clinical benefits, the high cost of the drug may hinder its widespread usage, especially for patients with limited economic areas. This represents a critical challenge in the clinical adoption of these therapies. To address these challenges, an evaluation of the economic evaluation of drugs has become imperative, as it provides a systematic approach to examine the costs and benefits in terms of different treatment options.

This study has several limitations that warrant consideration in healthcare decision-making. First, the clinical data included in the model have been obtained from the BEATcc trial, which may affect the results of the long-term survival model and increase model uncertainty. Second, the utility values were derived from published literature, some of the data were obtained from a foreign study, and bias was not distinguished due to different treatment strategies. Third, the cost values were collected from the published studies rather than the real world. However, there was an imbalance of economic development between different regions. Fourth, our study only included the costs of management SAEs (Grade≥3), and ignored the costs of AEs below grade 3, although these cost values only had minimal influence on the model results revealed by one-way sensitivity analyzes. Finally, we did not examine the economic outcomes in subgroups, such as the gender of the patients, which may have an impact on the results. Despite the limitations of the study, the sensitivity analysis demonstrated the robustness of our model outcomes. Therefore, it might provide valuable reference for clinical treatment decisions and policy-makers.

## 5. Conclusions

In conclusion, from the perspective of the Chinese health-care system, the addition of atezolizumab to chemotherapy and bevacizumab is not cost-effective compared to chemotherapy plus bevacizumab alone for patients with metastatic, persistent, or recurrent cervical cancer.

## Supporting information

S1 Table Summary of statistical goodness-of-fit of Kaplan-Meier curves in the BEATcc trial. * adopted parametric survival function in the model, AIC Akaike information criterion, BIC Bayesian information criterion, PFS progression-free survival, OS overall survival.
(DOCX)
