# Correction: Economic evaluation of atezolizumab in combination with bevacizumab and chemotherapy for metastatic, persistent, or recurrent cervical cancer in China: A cost-effectiveness analysis

**DOI:** 10.1371/journal.pone.0353864

**Published:** 2026-07-14

**Authors:** Shuo Yang, Yibing Hou, Xiaohui Wang, Shuo Kang, Zhenhua Pan

There is an error in affiliation 4 for author Shuo Kang. The correct affiliation 4 is: Medical Insurance Office, The Second Hospital of Hebei Medical University, Shijiazhuang, Hebei, People’s Republic of China.
