# Supplementary material for: Economic evaluation of atezolizumab in combination with bevacizumab and chemotherapy for metastatic, persistent, or recurrent cervical cancer in China: A cost-effectiveness analysis
Source: PLoS One. 2026 Jun 11;21(6):e0351029. doi: 10.1371/journal.pone.0351029 (PMC13257988; doi:10.1371/journal.pone.0351029)
Supplement: S1 Table — * adopted parametric survival function in the model, AIC Akaike information criterion, BIC Bayesian information criterion, PFS progression-free survival, OS overall survival. (DOCX) [file pone.0351029.s001.docx]

**Supporting information**

**S1 Table. Summary of statistical goodness-of-fit of Kaplan-Meier curves in the BEATcc trial.**

| Treatment | PFS | | OS | |
| --- | --- | --- | --- | --- |
|  | Bevacizumab plus chemotherapy | Atezolizumab plus bevacizumab and chemotherapy | Bevacizumab plus chemotherapy | Atezolizumab plus bevacizumab and chemotherapy |
| Exponential | 1225.215 | 1114.867 | 1140.955 | 992.7039 |
| Weibull | 1202.745 | 1109.912 | 1114.238 | 972.3034 |
| Gamma | 1192.915 | 1104.233 | 1114.365 | 971.7379 |
| Generalized gamma | 1183.274 | 1080.447 | 1115.978 | 973.7317 |
| Gompertz | 1223.397 | 1116.687 | 1123.502 | 979.886 |
| Lognormal | 1181.404 | 1082.768 | 1130.142 | 978.8156 |
| Log-logistic | 1174.398 | 1085.853 | 1115.048 | 971.2864 |

*** adopted parametric survival function in the model, *AIC* Akaike information criterion, *BIC* Bayesian information criterion, *PFS* progression-free survival, *OS* overall survival.
